# Supplementary material for: Advancing the scale of synthetic biology via cross-species transfer of cellular functions enabled by iModulon engraftment
Source: Nat Commun. 2024 Mar 15;15:2356. doi: 10.1038/s41467-024-46486-3 (PMC10943186; doi:10.1038/s41467-024-46486-3)
Supplement: Supplementary file 1 — Supplementary Information [file 41467_2024_46486_MOESM1_ESM.pdf]

Supplementary Information for

Advancing the scale of synthetic biology via cross-species transfer of cellular functions enabled by iModulon engraftment

Donghui Choe *et al*.

This PDF file includes:

Supplementary Notes — Section 1 and 2

Supplementary Tables 1 and 2

Supplementary Figs. 1 to 4

Supplementary References

SUPPLEMENTARY NOTES

Section 1. Repairing and reconfiguring broken branched-chain amino acid synthetic iModulons in *E. coli* K-12

Genes that are members of iModulons exist in trans-locations and transfer of all the genes on an iModulon requires refactoring into a contiguous piece of DNA. We refactored branched-chain amino acid (BCAA) biosynthetic iModulon in its native host. BCAAs are essential amino acids that play a critical role in protein synthesis and energy metabolism. The genes that constitute these biosynthetic functions are found in two iModulons in *E. coli* K-12 strains[1]. They can synthesize all three BCAAs — leucine, isoleucine, and valine — using intermediates from central carbon metabolism, pyruvate and oxaloacetate. The biosynthesis involves a series of enzymatic reactions, including the use of acetohydroxyacid synthase (AHAS) complexes. *E. coli* has three different AHAS isozymes, each with unique properties and product inhibition sensitivities[2,3]. This ensures the proper balance of production and utilization of the three BCAAs. However, the *ilvG* gene in *E. coli* K-12, which encodes a catalytic subunit of valine-insensitive AHAS II in conjunction with a regulatory subunit *ilvM,* has a frameshift mutation that renders the enzyme non-functional[4]. This mutation, combined with the inhibitory effects of valine on the other two AHAS isozymes, results in the failure of BCAA biosynthesis in the presence of excess valine[5]. In addition, the deficiency induces repeated isoleucine starvation in fermentation[6]. During our efforts to correct this genetic deficiency in the strain, we explored the potential of iModulon-based genome refactoring, with the goal of gaining insights that could be applied to the transfer of iModulons whose genes are in a trans-configuration on the genome. We are particularly interested in whether the presence of all the iModulon genes is sufficient and if the genomic organization, such as location, direction, and order of genes, is important for BCAA biosynthesis.

The iModulon structure of two closely-related *E. coli* K-12 strains MG1655 and BW25113, determined from more than 400 unique experimental conditions, shows that *E. coli* K-12 strains have two different iModulons dedicated to BCAA synthesis (**Supplementary Fig. 1A**)[1]. These iModulons encode multiple genes to synthesize BCAA from pyruvate or homoserine and complement each other due to the multifunctionality of some of the genes (**Supplementary Fig. 1B**). The two iModulons contain a full repertoire of genes required for the BCAA biosynthesis. Thus, we included the two iModulons in the reconfiguration of the complete BCAA biosynthetic pathways. The reconfiguration involves moving iModulon members from four different genomic loci to one locus altogether and aligning their orientation into the same direction. To set the boundary of transfer to preserve regulatory elements in the iModulon, we utilized transcription start sites (TSSs) and transcription termination sites (TTSs) represented by Bitomic information[5,7]. Outermost TSSs and TTSs that define the largest transcriptional unit were chosen for boundaries and DNA fragments containing the transcriptional unit were cloned and assembled (**Supplementary Fig. 1D**). During the cloning, a single nucleotide insertion was introduced to fix the frameshift mutation in *ilvG*. After removal of endogenous copies of all iModulon genes (ΔBCAA knockout strain) from the chromosome, the assembled iModulon construct was integrated into the chromosome at *thr* locus using replicon excision for enhanced genome engineering through programmed recombination (REXER)[8,9] (**Supplementary Fig. 1D**).

In an M9 glucose defined medium, the reconfigured strain (RECON) showed growth rates comparable to its parental strain, while the knock-out strain (ΔBCAA) failed to grow (**Supplementary Fig. 1E**). The knock-out strain was only able to grow with supplementation of all three BCAAs (**Supplementary Fig. 1E**). Wild-type MG1655 and the reconfigured strain behaved similarly with isoleucine and leucine supplementation (**Supplementary Fig. 1E**). With valine supplementation, the wild-type strain showed dramatic growth retardation due to the aforementioned valine toxicity (**Supplementary Fig. 1E**). The reconfigured strain showed no growth inhibition with external valine, indicating rewiring of BCAA biosynthesis. This reconfiguration demonstrates that the order, orientation, and location of genes on the genome had negligible effects on functionality if the refactored system had the complete set of genes. It suggests transferability of iModulons based on the transcriptional framework decoded by ICA[1].

Section 2. Metabolic failure induced by MdcR iModulon

The *E. coli* strain carrying the pMdcR_iM was unable to grow in M9 malonate medium when the governing promoter (IPTG-inducible Trc promoter) was induced. It is likely due to the inhibition of endogenous enzymes — succinate:quinone oxidoreductase[10] and isocitrate lyase[11] constituting TCA and glyoxylate cycle, respectively — by malonate. Suboptimal over expression of the iModulon would produce the malonate transporter MdcL and MdcM, increasing intracellular malonate levels, leading to a metabolic failure. We demonstrated that heterologous expression of iModulon could be rapidly optimized by ALE. During ALE, *polA* mutations occurred and the copy number of the plasmid was reduced, resulting in 4 to 50-fold decrease in *mdcA* expression. Besides, there was no mutation on the genes composing MdcR iModulon, which again indicates that iModulons are units of biological traits that require no extensive engineering at transfer.

SUPPLEMENTARY TABLES

Supplementary Table 1. Mutations occurred during the malonate ALE. Arrows indicate genetic orientation. Strain names are given as AX.IY. X is the ALE lineage number. Y is an arbitrary identifying number for the clonal isolate from the same ALE lineage. First five mutations are genetic variations observed in multiple isolates of the MG1655[12,13].

Location	Position	Gene	Mutation	Annotation	Mutant allele frequency						Description
					Parental	A1.I1	A2.I1	A3.I1	A3.I2	A3.I3	
Chromosome	257,908	*crl*::IS1I	Δ776 bp		1.000	1.000	1.000	1.000	1.000	1.000	pseudogene; RNA polymerase holoenzyme assembly factor
Chromosome	1,978,503	*insB1–insA*	Δ776 bp		1.000	1.000	1.000	1.000	1.000	1.000	insB1, insA
Chromosome	2,173,363	*gatC*	Δ2 bp	intergenic (-1/+1)	1.000	1.000	1.000	1.000	deleted	1.000	galactitol-specific PTS enzyme IIC component
Chromosome	3,560,455	*glpR* ← / ← *glpR*	+G	intergenic (-2/+1)	1.000	1.000	1.000	1.000	1.000	1.000	pseudogene, DNA-binding transcriptional repressor GlpR
Chromosome	4,296,381	*gltP* → / ← *yjcO*	+GC	intergenic (+587/+55)	0.926	0.973	1.000	0.929	1.000	1.000	glutamate/aspartate:proton symporter / Sel1 family TPR-like repeat protein
Chromosome	505,954	*ushA*	Δ1 bp	coding (1041/1653 nt)	0.000	0.000	0.000	0.000	0.989	0.000	5'-nucleotidase / UDP-sugar hydrolase
Chromosome	1,147,662	*plsX*	Δ2 bp::IS186(–)+6 bp	coding (42-47/1071 nt)	0.000	0.000	0.000	1.000	0.000	1.000	putative phosphate acyltransferase
Chromosome	1,354,286	*sapC*	A→G	L78P (CTG→CCG)	0.000	0.000	1.000	0.000	0.000	0.000	putrescine ABC exporter membrane protein
Chromosome	1,393,457	*mppA*	IS186(+)+5 bp	coding (231-235/1614 nt)	0.000	1.000	0.000	0.000	0.000	0.000	murein tripeptide ABC transporter periplasmic binding protein
Chromosome	1,888,687	*fadD*	G→A	L354L (CTG→TTG)	0.000	0.000	0.000	0.981	0.000	0.000	long-chain-fatty-acid—CoA ligase
Chromosome	1,901,632	*yoaE* ← / → *manX*	C→T	intergenic (-47/-416)	0.000	0.000	0.000	0.000	0.986	0.000	TerC family inner membrane protein / mannose-specific PTS enzyme IIAB component
Chromosome	2,167,510	*ogrK–fbaB*	Δ11,303 bp		0.000	0.000	0.000	0.000	0.974	0.000	*ogrK, yegZ, yegR, yegS, gatR, insE1, insF1, gatR, gatD, gatC, gatC, gatB, gatA, gatZ, gatY, fbaB*
Chromosome	2,725,578	*kgtP*	Δ1 bp	coding (169/1299 nt)	0.000	0.980	0.000	0.000	0.000	0.000	alpha-ketoglutarate transporter
Chromosome	3,213,681	*rpoD*	Δ6 bp	coding (635-640/1842 nt)	0.000	0.000	0.000	1.000	0.966	1.000	RNA polymerase, sigma 70 (sigma D) factor

Chromosome	3,437,535	*trkA*	C→A	Q340K (CAG→AAG)	0.000	0.000	0.000	0.000	1.000	0.000	NAD-binding component of TrK potassium transporter
Chromosome	3,676,881	*yhjG*	C→A	M490I (ATG→ATT)	0.000	0.000	0.000	0.981	1.000	0.985	putative inner membrane-anchored periplasmic AsmA family protein
Chromosome	3,815,808	*pyrE* ← / ← *rph*	$(A)_{8\rightarrow7}$	intergenic (-40/+26)	0.000	0.000	1.000	0.000	0.000	0.000	orotate phosphoribosyltransferase / ribonuclease PH (defective)
Chromosome	4,047,393	*polA*	A→C	Q143P (CAG→CCG)	0.000	1.000	0.000	0.000	0.000	0.000	DNA polymerase I
Chromosome	4,047,417		T→G	L151R (CTT→CGT)	0.000	0.000	0.000	0.993	0.974	0.983	
Chromosome	4,048,959		C→A	A665E (GCA→GAA)	0.000	0.000	1.000	0.000	0.000	0.000	
Plasmid	No mutation was found from the pMdcR_iM plasmid DNA.										

Supplementary Table 2. Primers used in this study.

Experiment	Primer name	Primer sequence (5' to 3')
Construction of BAC_BCAA_iM	BAC_frag1_F	TTTTTATGAAGAAATTATGGAGAAAAATGACAGGGAAAAAGGAGAAATTCTCAATAAATGCGGTAACTTACCTAGGAAGCTTGAGCACGT
	BAC_frag1_R	GTGGCACTTTTCGGGGAAATGTGCGCGGAACCCCTATTTGTTTTGTCCACATAACCGTGC
	BAC_frag2_F	GCTTATCCACAACATTTTGCGCACGGTTATGTGGACAAAACAAATAGGGGTTCCGCGCAC
	BAC_frag2_R	GACATATTGCCCGTTGCAGTCAGAATGAAAAGCTCCTAGGCCACTATTTATACCATGGGA
	RECON_PK_F	ATCAAGATCTGATCAAGAGACAGGATGAGGATCGTTTCGCATGAGCCACTTAGCTGAGTT
	RECON_PK_R	TAAGTTACCGCATTTATTGAGAATTTCTCCTTTTTCCCTGTCATTTTTCTCCATAATTTCTTCATAAAAATCAGAAGAACTCGTCAAGAA
	RECON_Pkan_F	TGAATTAATCCCCTTGCCCGGTCAAATGACCGGGCTTTCCAGAGCGCTTTTGAAGCTCAC
	RECON_Pkan_R	CGGCTTTGGCTGATGCTACTAACTCAGCTAAGTGGCTCATGCGAAACGATCCTCATCCTG
	RECON_Thr_F	CTCGGTTTGACGCCTCCCATGGTATAAATAGTGGCCTAGGAGCTTTTCATTCTGACTGCA
	RECON_Thr_R	TAAAAATAAAATAGTGAGTGAAAAAAATTCAATTTCAGTAAAAAAAGCCGGGCTGCATAA
	RECON_ilvG_F	AGGCCGGGTTTGCTTTTATGCAGCCCGGCTTTTTTTACTGAAATTGAATTTTTTTCACTC
	RECON_ilvG_R	TCACGCAGCTGCGCGCAGTGTTGCTGCCAGTCATTGATATTTAACGGCTGCTGTAATGCT
	RECON_ilvMEDA_F	TTTAAATGCTCTGTTACCAGCATTACAGCAGCCGTTAAATATCAATGACTGGCAGCAACA
	RECON_ilvMEDA_R	TTGCGGAAATTGATATATTCACAACGTCACATTGCAATTAAAAAGCGGCTACATGAGCCG
	RECON_ilvC_F	TCCAGAATGCCTCAATTAGCGGCTCATGTAGCCGCTTTTTAATTGCAATGTGACGTTGTG
	RECON_ilvC_R	AATTAATGCATTAAATATATAAATTAATTATTAAATAAGCTAGCAAGGCCTTCTCCAGGA
	RECON_Leu_F	AAACACTCAAGGCCTTCTCCTGGAGAAGGCCTTGCTAGCTTATTTAATAATTAATTTATATATTTAATGC
	RECON_Leu_R	GCGCCCTGAGTGCTTGCGGCAGCGTGAGCTTCAAAAGCGCTCTGGAAAGCCCGGTCATTT
Construction of ΔBCAA strain carrying sacB-cat dual-selection landing pad	ilv_KO_F	CAAAAATGCAGCGGACAAAGGATGAACTACGAGGAAGGGAACAACATTCAGTGTAGGCTGGAGCTGCTTC
	ilv_KO_R	TGCACACCCAGTTCGGATATCACATCATTAAGGTGCTGTACCGCAACTAAGGGATCCGTCGACCTGCAGT
	ilvY_PK_HF	CAAAAATGCAGCGGACAAAGGATGAACTACGAGGAAGGGAACAACATTCAAAAAATGCCTGATAGCGCTT
	ilvY_PK_JR	CTTCAAAAGCGCTCTAGGAAATTTATTGCGGAAATTG

	ilvY_PK_JF	CGCAATAAATTTCCTAGAGCGCTTTTGAAGCTC
	ilvY_PK_HR	TGCACACCCAGTTCGGATATCACATCATTAAGGTGCTGTACCGCAACTAATCAGAAGAACTCGTCAAGAA
	delPK1_F	GCCAAAATGGCGGCTTT
	delPK1_R	CTGTACCGCAACTAAAGGAAATTTATTGCGGAAATTGA
	delPK2_F	CCGCAATAAATTTCCTTTAGTTGCGGTACAGCACCT
	delPK2_R	CGGCGGTGATTTAGGTGAA
	Leu_KO_F	ATTATTTTTAACCGCAAAGGTTAAAGACGTTTGATGACGTGGACGATAGCGTGTAGGCTGGAGCTGCTTC
	Leu_KO_R	TAATCATAACTACCGCGAATACTCAATCATCTACAAAATGGATTAAATGTGGGATCCGTCGACCTGCAGT
	SC_F	AGCTTTTCATTCTGACTGCAACGGGCAATATGTCTCTGTGTGGATTAAAAAAAGAGTGTCTGATAGCAGCCTAAGGTTGACAATTAATCATCGGCATAG
	SC_R	TTGGTTAAAGTATTTAGTGACCTAAGTCAATAAAATTTTAATTTACTCACGGCAGGTAACCAGTTCAGAAACGTGCTCAAGCTTCCTAGG
Construction of spacer plasmid	RX4_1_F	GAAATTCTCAATAAATGCGGTAACTTACCTGTTTTAGAGCTATGCTGTTTTGAATGGTCCCAAAACATTAAAAAAAGAGTGTCTGATAGCAGCCTA
	RX4_1_R	TAGGCTGCTATCAGACACTCTTTTTTTAATGTTTTGGGACCATTCAAAACAGCATAGCTCTAAAACAGGTAAGTTACCGCATTTATTGAGAATTTC
	RX4_2_F	GACAAATAGTGCGATTACGAAATTTTTTAGACAAAAATAGTCTACGAGGTTTTAGAGCTATGCTGTTTTGAATGGTCCCAAAACTGCCCGTTGCAGTCAGAATGAAAAGCTCCTGTTTTAGAGCTATGCTGTTTTGAATGGTCCCAAAACGAAATTCTCAATAAATGCGGTAACTTACCT
	RX4_2_R	GATACTGAGCACATCAGCAGGACGCACTGACCGAATTCAACTCAACAAGTCTCAGTGTGCTGAAGTTTTGGGACCATTCAAAACAGCATAGCTCTAAAACTTTTTATGAAGAAATTATGGAGAAAAATGAGTTTTGGGACCATTCAAAACAGCATAGCTCTAAAACTAGGCTGCTATCAGACACTCTTTTTTTAAT
	pUC_spacer_F	CTGCTGATGTGCTCAGTATC
	pUC_spacer_R	TCGTAATCGCACTATTTGTC
Amplification of spacer array	Spacer_amp_F	AATAGGCGTATCACGAGGC
	Spacer_amp_R	ACCGTATTACCGCCTTTGAG
pVanR_iM	pTrc_inv_F	GTTTAAACGGTCTCCAGCTT
	pTrc_inv_R	GGTTTATTCCTCCTTATTTAATCG
	vanA_F	TAAATAAGGAGGAATAAACCATGTACCCCAAAAACACCTGG
	vanB_R	ACCTGCCGGCCAGGCTCAGATGTCCAGCACCAGCA
	vanK_F	GTGCTGGACATCTGAGCCTGGCCGGCAGGTG
	vanK_R	TTGTTCTCCACGTTCTCAGTGGCTCAGTGCATCAG
	galP_F	TGCACTGAGCCACTGAGAACGTGGAGAACAATAATAATGA
	galP_R	AAGCTGGAGACCGTTTAAACCTACAAGGTCAGCGGATAG
pMdcR_iM	mdcR_iM_F	TAAATAAGGAGGAATAAACCATGACGACACCGATCTCTCCCC

	mdcR_iM_R	AAGCTGGAGACCGTTTAAACTCACCCAACCAGGCCGCG
pOp353	PP_Op353_F	TAAATAAGGAGGAATAAACCATGCCGCTACCCGCCC
	PP_Op353_R	AAGCTGGAGACCGTTTAAACTTATTAGATCCTGCCCAACCTGCG
pAcoR_iM	PP_bdhA_R	AAGCTGGAGACCGTTTAAACTTATTAGCGCACACCTGGTGAAACG
pBDO_pathway	PP_acoA_F	AAGCTGGAGACCGTTTAAACTTATTAGCGCACACCTGGTGAAACG
BAC_AmpC_iM	ampC_1_F	TATTAATGTATCGATTAAATAAGGAGGAATAAACCATGCGCGATACCAGATTCCCCTGC
	ampC_1_R	CTCTGTTGTACGTTCACCCGATATTCCGTCCCTGCGCTCAGGCGTCGCGCAGCAG
	PA4112_2_F	CCCTGCTCAACGCCCATGACCTGCTGCGCGACGCCTGAGCGCAGGGACGGAATATCG
	PA4112_2_R	GGCCAGCGTGCGGTTCATGGGGGACTCTCCGTCGGGGTTCAGCTCGCCGACTCCGC
	creD_3_F	GATGTTCGGCGACGTGCAGGCGGAGTCGGCGAGCTGAACCCCGACGGAGAGTCCC
	creD_3_R	GTATGGGTAGCGCTCATGCGGGCTCCTTGGAAGGATCTCATTTCCAGCACACCAGGC
	PA0467_4_F	TGGGCCTGCTGCTGTTCCGCCTGGTGTGCTGGAAATGAGATCCTTCCAAGGAGCCCGCATG
	PA0467_4_R	CAGGTGACGAAGTTTCATGGGTAACGCCTCGTAGTTGGTCAGGCGCGGGTGGCCTG
	PA0320_5_F	CAGCCAGCGCCCGTCGATGCAGGCCACCCGCGCCTGACCAACTACGAGGCGTTAC
	PA0320_5_R	CTGAGTGCTTGCGGCAGCGTGAGCTTCAAAAGCGCTCTTTACTTGATCACTTCGACG
	PK_F	CCGCGAGATCGACGTCGAGTTCGTCGAAGTGATCAAGTAAAGAGCGCTTTTGAAGCTCAC
	PK_R	TAAGTTACCGCATTTATTGAGAATTTCTCCTTTTTCCCTGTCATTTTTCTCCATAATTTCTTCATAAAAATCAGAAGAACTCGTCAAGAA
	BAC_AmpC_iM_1_F	TTTTTATGAAGAAATTATGGAGAAAAATGACAGGGAAAAAGGAGAAATTCTCAATAAATGCGGTAACTTACCTAGGAAGCTTGAGCACGT
	BAC_AmpC_iM_1_R	GTGGCACTTTTCGGGGAAATGTGCGCGGAACCCCTATTTGTTTTGTCCACATAACCGTGC
	BAC_AmpC_iM_2_F	GCTTATCCACAACATTTTGCGCACGGTTATGTGGACAAAACAAATAGGGGTTCCGCGCAC
	BAC_AmpC_iM_2_R	CAATTCGCGCTAACTCACATTAATTGCGTTGCGCCCACTATTTATACCATGGGA
	Ptrc_F	CAAAAACTCGGTTTGACGCCTCCCATGGTATAAATAGTGGGCGCAACGCAATTAATGTGA
	Ptrc_R	CGGCGATGCCGCACAGGCAGGGGAATCTGGTATCGCGCATGGTTTATTCCTCCTTATTTAATC
BAC_ampC	ampC_only_R	CGCCCTGAGTGCTTGCGGCAGCGTGAGCTTCAAAAGCGCTCTTCAGCGCTTCAGCGGCAC

	ampC_only_Ptrc_F	CGGCCTGGAGCAGCAGGGCAAGGTGCCGCTGAAGCGCTGAAGAGCGCTTTTGAAGCTCAC
P/C ratio (qPCR)	bla_F	ATTATCCCGTGTTGACGCCG
	bla_R	TTCGGTCCTCCGATCGTTGT
	alaA_F	ACACGCCAAAGGCTACATCG
	alaA_R	ACGACCGCCAGGGGTAATAA
mdcA expression level (qRT-PCR)	mdcA_qRT_F	CTCGACTTCTCCTTCTCCGG
	mdcA_qRT_R	ACGTTGGGGATCAGGTCG
	rrsA_qRT_F	CGATCCCTAGCTGGTCTGAG
	rrsA_qRT_R	TTCTTCATACACGCGGCATG

SUPPLEMENTARY FIGURES

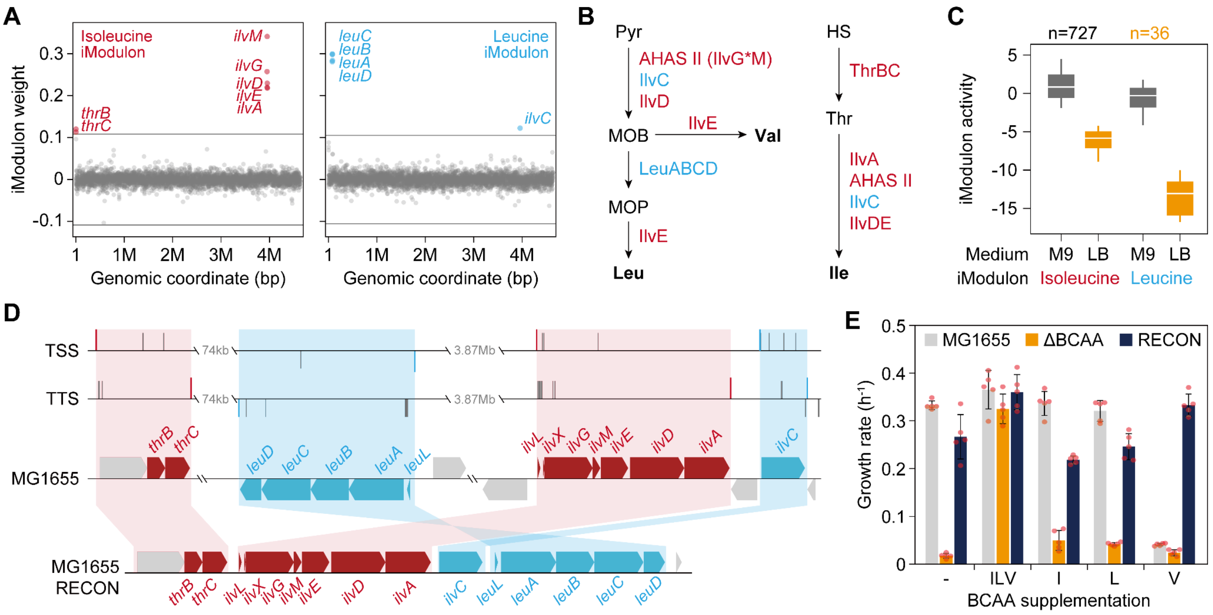

Supplementary Figure 1. Reconfiguration and repair of branched-chain amino acids (BCAA) biosynthetic iModulons in *E. coli* K-12. (**A**) Scatter plots show iModulon weights of genes contained in isoleucine and leucine biosynthetic iModulons. Horizontal gray lines indicate thresholds for determining iModulon membership. (**B**) The BCAA biosynthetic pathway in *E. coli* MG1655. *ilvG* carries a frameshift mutation inactivating the gene. Enzymes from Isoleucine and Leucine iModulons are colored red and blue, respectively. MOB, 3-methyl-2-oxobutanoate. MOP, 4-methyl-2-oxopentanoate. HS, homoserine. (**C**) Activities of isoleucine and leucine iModulons in M9 defined medium (gray) or LB medium (yellow). The PRECISE 1K dataset is centered on M9 thus the iModulon activities are near zero under these conditions, but deactivated in LB. Box limits, whiskers, and center lines indicate 1st and 3rd quartiles, 10 and 90 percentiles, and median of the distribution, respectively. (**D**) Genomic landscape of genes in the BCAA iModulons in the MG1655 strain and their reconfigured structure (RECON). Transcription start sites (TSSs) and transcription termination sites (TTSs) guide the border of genetic rearrangement. Genes from Isoleucine and Leucine iModulons are colored red and blue, respectively. Shades indicate genetic rearrangements. (**E**) Growth rates of *E. coli* MG1655 (gray bars), BCAA biosynthesis knock-out (ΔBCAA, orange bars), and BCAA reconfigured (RECON, blue bars) strains in M9 glucose, M9 glucose supplemented with all three BCAA (ILV), isoleucine (I), leucine (L), or valine (V). Data are presented as mean values +/− SD. Error bars indicate SD of five biological replicates. Circles show individual data points.

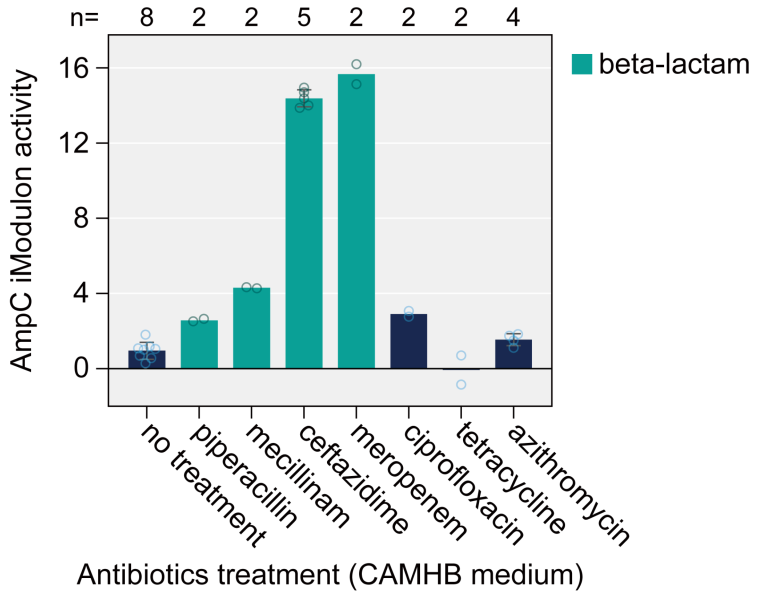

Supplementary Figure 2. Activity of AmpC iModulon in response to antibiotics treatment. Data are presented as mean values +/− SD. Error bars indicate SD of the sample set (when n>2). Circles represent individual transcriptome samples.

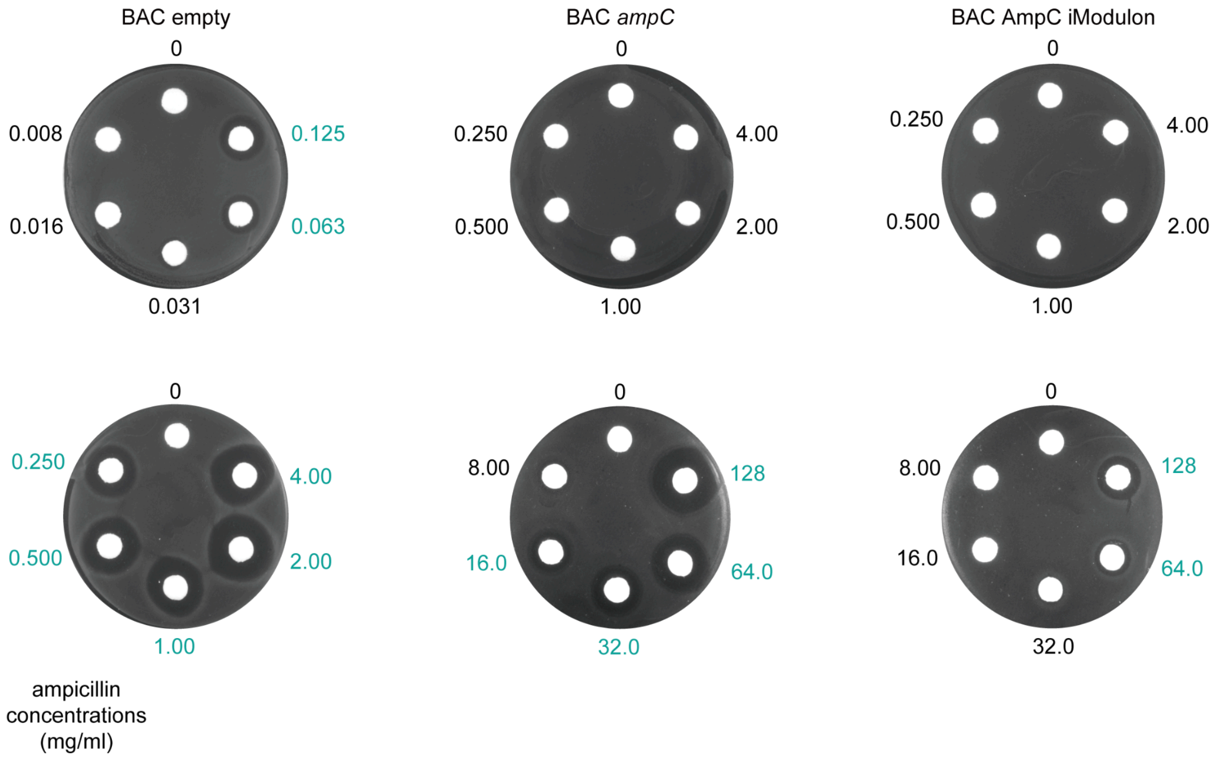

Supplementary Figure 3. Ampicillin disc diffusion assay of *E. coli* carrying empty plasmid, *P. aeruginosa ampC,* or AmpC iModulon. Numbers indicate concentration of ampicillin added to the paper disk. Disks with inhibitory halos are marked as green colored numbers.

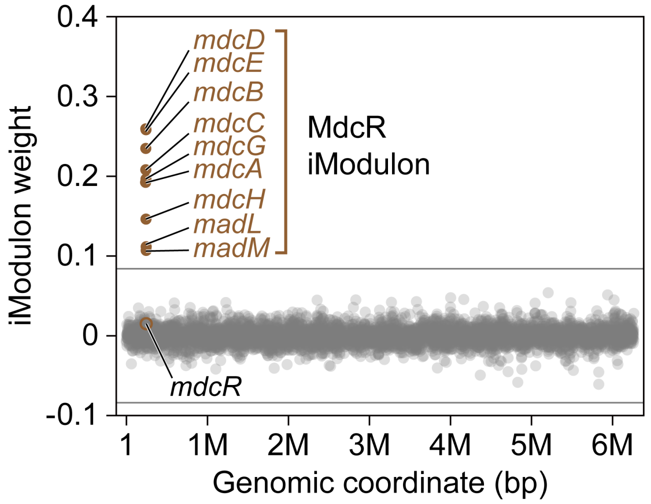

Supplementary Figure 4. MdcR iModulon weights of genes in *P. aeruginosa*. Nine genes constitute the MdcR iModulon (filled brown circles). Gray circles identify genes not in the iModulon. Gray lines indicate iModulon weight threshold.

SUPPLEMENTARY REFERENCES

1. Lamoureux, C. R. *et al.* A multi-scale expression and regulation knowledge base for *Escherichia coli*. *Nucleic Acids Res.* (2023) doi:10.1093/nar/gkad750.
2. Blatt, J. M., Pledger, W. J. & Umbarger, H. E. Isoleucine and valine metabolism in *Escherichia coli*. XX. Multiple forms of acetohydroxy acid synthetase. *Biochem. Biophys. Res. Commun.* **48**, 444–450 (1972).
3. Vinogradov, V. *et al.* Acetohydroxyacid synthase isozyme I from *Escherichia coli* has unique catalytic and regulatory properties. *Biochim. Biophys. Acta* **1760**, 356–363 (2006).
4. Lawther, R. P. *et al.* Molecular basis of valine resistance in *Escherichia coli* K-12. *Proc. Natl. Acad. Sci. U. S. A.* **78**, 922–925 (1981).
5. Choe, D. *et al.* Adaptive laboratory evolution of a genome-reduced *Escherichia coli*. *Nat. Commun.* **10**, 935 (2019).
6. Andersen, D. C., Swartz, J., Ryll, T., Lin, N. & Snedecor, B. Metabolic oscillations in an *E. coli* fermentation. *Biotechnol. Bioeng.* **75**, 212–218 (2001).
7. Lamoureux, C. R. *et al.* The Bitome: digitized genomic features reveal fundamental genome organization. *Nucleic Acids Res.* **48**, 10157–10163 (2020).
8. Wang, K. *et al.* Defining synonymous codon compression schemes by genome recoding. *Nature* **539**, 59–64 (2016).
9. Robertson, W. E. *et al.* Creating custom synthetic genomes in *Escherichia coli* with REXER and GENESIS. *Nat. Protoc.* **16**, 2345–2380 (2021).
10. Maklashina, E. & Cecchini, G. Comparison of catalytic activity and inhibitors of quinone reactions of succinate dehydrogenase (Succinate-ubiquinone oxidoreductase) and fumarate reductase (Menaquinol-fumarate oxidoreductase) from *Escherichia coli*. *Arch. Biochem. Biophys.* **369**, 223–232 (1999).
11. Hoyt, J. C., Robertson, E. F., Berlyn, K. A. & Reeves, H. C. *Escherichia coli* isocitrate lyase: properties and comparisons. *Biochim. Biophys. Acta* **966**, 30–35 (1988).
12. Freddolino, P. L., Amini, S. & Tavazoie, S. Newly identified genetic variations in common *Escherichia coli* MG1655 stock cultures. *J. Bacteriol.* **194**, 303–306 (2012).
13. Khare, A. & Tavazoie, S. Multifactorial Competition and Resistance in a Two-Species Bacterial System. *PLoS Genet.* **11**, e1005715 (2015).
